# Persistence of Symptoms among Commercially Insured Patients with Coccidioidomycosis, United States, 2017–2023

**DOI:** 10.3201/eid3114.250022

**Published:** 2025-12

**Authors:** Ian Hennessee, Samantha L. Williams, Kaitlin Benedict, Dallas J. Smith, George R. Thompson, Mitsuru Toda

**Affiliations:** Centers for Disease Control and Prevention, Atlanta, Georgia, USA (I. Hennessee, S.L. Williams, K. Benedict, D.J. Smith, M. Toda); University of California, Davis, Sacramento, California, USA (G.R. Thompson III)

**Keywords:** fungi, Coccidioidomycosis, Valley fever, signs and symptoms, postinfectious disorders, fatigue, infection-associated chronic illnesses and conditions, United States

## Abstract

Some patients with coccidioidomycosis experience prolonged respiratory and systemic symptoms. However, data on prevalence and persistence of most symptoms are lacking. Using an insurance claims database, we identified patients with coccidioidomycosis diagnoses in the United States during 2017–2023. We assessed prevalence of associated symptoms from 6 months before to 1 year after first diagnosis code (index date) and compared post–index date prevalence to baseline (within 6 to 4 months before index date). Among 2,640 patients, cough (20.8%), dyspnea (13.0%), and fatigue (8.8%) were the most common symptoms at index date. Dyspnea and erythema nodosum were elevated 3–6 months post–index date (p<0.03), and fatigue, headache, joint pain, and weakness were elevated 9–12 months post–index date compared with baseline (p<0.05).These findings demonstrate that symptoms can persist in coccidioidomycosis patients, which could help inform clinical management and refine estimates of the health and economic burden of coccidioidomycosis.

Coccidioidomycosis is a fungal infection that often presents as acute pulmonary infection; symptoms include cough, fatigue, dyspnea, and fever (*1*–*3*). A small percentage of those affected experience chronic pulmonary or disseminated disease (*4*).

Although coccidioidomycosis symptoms are often self-limited, some might persist for months or years (*5*). In previous studies, patients have self-reported median symptom duration of 60–120 days (*1*,*2*). Fatigue can be particularly long-lasting and can persist after all other evidence of the infection has resolved (*3*,*4*,*6*,*7*). Prolonged symptoms of coccidioidomycosis can cause substantial negative effects; evidence suggests that nearly three-quarters of patients are limited in their ability to perform usual daily activities for a median of 40–47 days during their illness (*1*,*2*).

Although chronic fatigue is increasingly recognized as a characteristic feature of coccidioidomycosis (*6*), data on the persistence of other symptoms are limited. Such information could help inform clinical management and refine estimates of the health and economic burden of coccidioidomycosis. We describe the prevalence and persistence of symptoms among patients with coccidioidomycosis by using a large US health insurance claims database.

## Methods

We used the Merative MarketScan Commercial/Medicare Database (https://www.merative.com/documents/brief/marketscan-explainer-general), which includes health insurance claims data for >48 million patients with employer-sponsored plans, including Medicare Supplemental and Medicare Advantage plans, throughout the United States during July 31, 2017–January 31, 2023. We identified patients with coccidioidomycosis diagnoses using International Classification of Diseases, 10th Revision, Clinical Modification (ICD-10-CM) codes (Appendix Table 1). Included patients had continuous insurance enrollment in the 180 days before and 365 days after the date the coccidioidomycosis diagnosis code was first used (index date) during the study window. We excluded patients for whom the coccidioidomycosis diagnosis code was listed on a laboratory or imaging claim alone, to minimize diagnoses recorded because the healthcare provider was attempting to rule out coccidioidomycosis. To capture incident diagnoses, we excluded patients with a coccidioidomycosis diagnosis code within the 6 months before the index date.

We assessed the prevalence of symptoms associated with coccidioidomycosis, which include abnormal weight loss, chest pain, chills without fever, cough, erythema nodosum or multiforme, fatigue or malaise, fever, generalized hyperhidrosis, headache, myalgia, joint pain, dyspnea, and weakness (*4*,*8*). We also examined the prevalence of a subset of symptoms and conditions associated with other infection-associated chronic illnesses and conditions (IACCIs): depression, dizziness, general paresis, generalized anxiety disorder, hypoactive sexual desire, insomnia, irritable bowel syndrome, palpitations, sleep apnea, and tinnitus (https://www.cdc.gov/chronic-symptoms-following-infections/about/index.html). We assessed the period prevalence of each symptom and condition (for brevity, hereafter referred to as symptoms) during nonoverlapping 30-day periods, from 6 months before to 1 year after index date.

To evaluate the persistence of symptoms, we next used log-binomial regression to compare the prevalence of each symptom within a baseline period before coccidioidomycosis illness (180–121 days before the index date) with the prevalence of symptoms within 4 consecutive 3-month follow-up periods after the index date: 0–89 days (0–3 months), 90–179 days (3–6 months), 180–269 days (6–9 months), and 270–365 days (9–12 months). We selected the baseline period on the basis of previous data indicating that in >90% of patients with coccidioidomycosis, the disease is diagnosed within 120–180 days of illness onset (*9*,*10*).

Finally, we assessed potential factors associated with symptom trends in patients with coccidioidomycosis: presence of underlying medical conditions (asthma or chronic obstructive pulmonary disease, diabetes, or immunosuppression), sex, age group (<18, 18‒44, 45‒64, or >65 years of age), receipt of fluconazole (>30-day supply, representing a clinically significant duration), and coccidioidomycosis diagnosis type (pulmonary, extrapulmonary, other, or unspecified). We used log-binomial regression to compare symptom prevalence across the levels of each factor (e.g., comparing patients with underlying conditions to patients without) within baseline and the 4 follow-up periods as described. We calculated p values by using the Wald test. We conducted analyses using the Merative MarketScan Treatment Pathways analysis tool (Merative) and R version 4.4.0 (The R Project for Statistical Computing, https://www.r-project.org). 

This activity was reviewed by the Centers for Disease Control and Prevention (CDC) and was conducted consistent with applicable federal law and CDC policy (e.g., 45 C.F.R. part 46.102(l)(2), 21 C.F.R. part 56; 42 U.S.C. §241(d); 5 U.S.C. §552a; 44 U.S.C. §3501 et seq.). Because MarketScan data are fully deidentified, this analysis was not subject to review by the CDC institutional review board.

## Results

Among 6,062 patients with coccidioidomycosis during the study window, we identified 3,113 (51%) who met the continuous insurance enrollment criteria. We excluded 474 (15%) persons who had a previous coccidioidomycosis diagnosis in the 6 months before the index date, leaving 2,640 coccidioidomycosis patients in the analytic cohort (Table 1). Among all patients, 52% were male and 48% were female, most were between the ages of 18‒44 (30%) or 45‒64 (50%), and most lived in the West (78%) and in nonrural areas (95%). Approximately 66% had documented underlying conditions. Pulmonary coccidioidomycosis (51%) and other or unspecified coccidioidomycosis (48%) were the most common coccidioidomycosis diagnoses on the index date.

**Table 1:** Demographic characteristics, underlying conditions, and coccidioidomycosis type among 2,640 commercially insured patients with coccidioidomycosis, United States, July 2017‒January 2023*

Characteristic	No. (%)
Sex	
M	1,363 (52)
F	1,277 (48)
Median age, y (IQR)	51.0 (39.0–60.0)
Age group, y	
<18	123 (5)
18‒44	803 (30)
45‒64	1,326 (50)
>65	388 (15)
US Census region of primary beneficiary's residence†	
Northeast	80 (3)
Midwest	219 (8)
South	266 (10)
West	2,047 (78)
Rural status‡	
Rural	123 (5)
Nonrural	2,304 (95)
Underlying conditions	898 (66)
Asthma or COPD	633 (24)
Diabetes	426 (18)
Hypertension	850 (35)
Immunosuppression§	971 (40)
Coccidioidomycosis type on index date	
Pulmonary	1,336 (51)
Cutaneous	52 (2)
Disseminated	118 (4)
Other or unspecified	1,277 (48)

*Values are no. (%) except as indicated. COPD, chronic obstructive pulmonary disease; IQR, interquartile range. †Regions were derived from US Census regions (https://www2.census.gov/geo/pdfs/maps-data/maps/reference/us_regdiv.pdf). ‡Rural versus nonrural status were based on US Census metropolitan statistical area designation. §Immunosuppression related to autoimmune disease, cancer, HIV, solid organ transplant, immunosuppressive medication, or a combination of those factors.

### Period Prevalence of Symptoms

**Figure 1:**
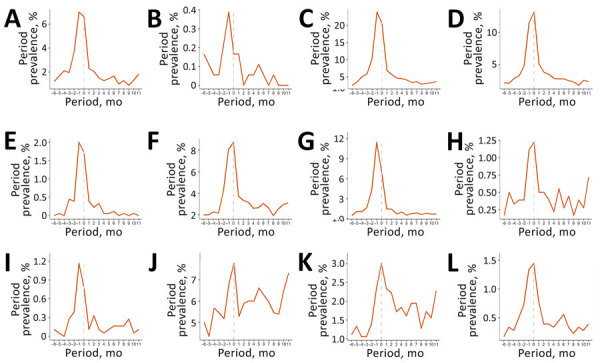
Period prevalence of selected coccidioidomycosis symptoms among commercially insured patients with coccidioidomycosis, United States, July 2017‒January 2023. Prevalence is shown for chest pain (A), chills (B), cough (C), dyspnea (D), erythema nodosum or multiforme (E), fatigue (F), fever (G), headache (H), hyperhidrosis (I), joint pain (J), weakness (K), and weight loss (L). The index period (0–29 days after index date) is shown with a dotted line. Data for myalgia are not shown because period prevalence was <1%. X-axis labels represent the beginning of 1-month follow-up periods relative to the index date (e.g., –6 refers to the period of 6–5 months before the index date).

**Figure 2:**
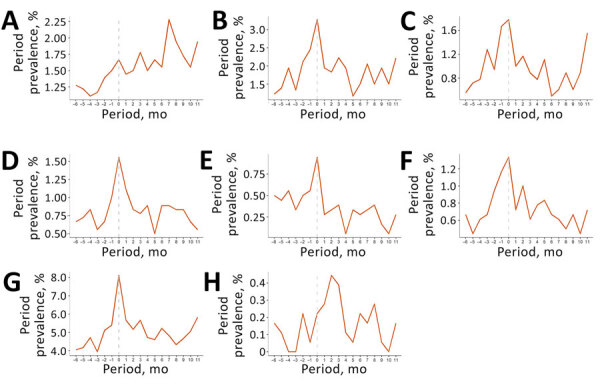
Period prevalence of selected symptoms from infection-associated chronic conditions and illnesses among commercially insured patients with coccidioidomycosis, United States, July 2017‒January 2023. Prevalence is shown for anxiety (A), depression (B), dizziness (C), insomnia (D), irritable bowel syndrome (E), palpitations (F), sleep apnea (G), and tinnitus (H). The index period (0–29 days after index date) is shown with a dotted line. Anger, hypoactive sexual desire disorder, and paresis are not shown because prevalence was <0.1% in all periods. X-axis labels represent the beginning of 1-month follow-up periods relative to the index date (e.g., –6 refers to the period of 6–5 months before the index date).

Among patients in the cohort, most symptoms increased in the months preceding the index date and peaked during the index period (0–29 days after index date) (Figure 1). During the index period, cough (20.8%), dyspnea (13.0%), and fatigue (8.8%) were the most prevalent coccidioidomycosis-associated symptoms. Most IACCI-associated symptoms also increased in the months preceding the index date and peaked during the index period, although anxiety continued to increase after the index date (Figure 2; Appendix Tables 2, 3). Sleep apnea was the most common IACCI-associated symptom at index date (8%).

The period prevalence of almost every symptom was higher at 0–3 months after the index date than at baseline (Table 2). Most symptoms then declined in the following periods (Figures 1, 2), although many remained elevated compared with baseline levels. Dyspnea and erythema nodosum or multiforme remained elevated 3–6 months after index date (p<0.03) (Table 2). Fatigue, joint pain, and weakness were elevated in all follow-up periods and remained higher 9–12 months after the index date than at baseline (p<0.05). Headache was also higher at 0–3 months and 9–12 months after index date than at baseline (p<0.03). The prevalence of most IACCI-related symptoms was similar to baseline by 3–6 months after index date, but dizziness was marginally higher among patients 9–12 months post–index date compared with baseline (p<0.10). Anxiety prevalence was not significantly different from baseline at 0–3 months post–index date but was marginally elevated 6–9 months and 9–12 months post–index date compared with baseline (p<0.08).

**Table 2:** Prevalence ratios of symptoms among commercially insured patients with coccidioidomycosis during 4 follow-up periods after index date compared with baseline, United States, July 2017‒January 2023*

	0–3 months			3–6 months			6–9 months			9–12 months	
Characteristic	PR (95% CI)	p value		PR (95% CI)	p value		PR (95% CI)	p value		PR (95% CI)	p value
Coccidioidomycosis-associated symptoms											
Abnormal weight loss	3.64 (2.08–6.82)	<0.001		1.29 (0.64–2.63)	0.5		1.21 (0.60– 2.50)	0.6		1.29 (0.64– 2.63)	0.5
Chest pain	2.10 (1.71–2.60)	<0.001		0.92 (0.71–1.18)	0.5		0.69 (0.53– 0.91)	0.009		0.83 (0.64– 1.07)	0.15
Cough	2.83 (2.48–3.24)	<0.001		1.16 (0.99–1.37)	0.063		0.89 (0.75–1.06)	0.2		0.92 (0.77–1.09)	0.3
Dyspnea	3.05 (2.56–3.67)	<0.001		1.29 (1.05–1.59)	0.018		1.10 (0.89–1.37)	0.4		0.90 (0.72–1.14)	0.4
Erythema nodosum or multiforme	28.6 (6.97–117)	<0.001		5.50 (1.48–35.5)	0.026		2.50 (0.54–17.4)	0.3		1.50 (0.25–11.4)	0.7
Fatigue or malaise	2.48 (2.06–3.00)	<0.001		1.31 (1.06–1.62)	0.012		1.33 (1.08–1.65)	0.008		1.29 (1.04–1.60)	0.019
Fever	3.28 (2.56–4.24)	<0.001		0.88 (0.64–1.22)	0.4		0.67 (0.47–0.95)	0.026		0.76 (0.54– 1.07)	0.12
Generalized hyperhidrosis	4.43 (2.07–10.9)	<0.001		1.57 (0.62– 4.26)	0.3		1.00 (0.34– 2.92)	>0.9		1.00 (0.34– 2.92)	>0.9
Headache	2.55 (1.58–4.24)	<0.001		1.50 (0.88– 2.60)	0.14		1.14 (0.64– 2.03)	0.7		1.82 (1.09– 3.10)	0.024
Myalgia	2.42 (1.27–4.91)	0.010		1.25 (0.59– 2.72)	0.6		1.00 (0.45– 2.25)	>0.9		0.50 (0.17– 1.29)	0.2
Pain in joint	1.28 (1.10–1.50)	0.001		1.19 (1.02– 1.40)	0.027		1.17 (1.00– 1.37)	0.050		1.25 (1.07– 1.46)	0.005
Weakness	2.53 (1.85–3.51)	<0.001		1.49 (1.05– 2.13)	0.026		1.71 (1.22– 2.42)	0.002		1.86 (1.34– 2.62)	<0.001
IACCI symptoms											
Depression	1.59 (1.22–2.07)	<0.001		1.17 (0.89–1.56)	0.3		1.18 (0.90–1.57)	0.2		1.15 (0.87–1.53)	0.3
Dizziness	1.73 (1.23–2.47)	0.002		1.10 (0.75–1.62)	0.6		0.96 (0.64–1.43)	0.8		1.37 (0.95–1.98)	0.092
Generalized anxiety disorder	1.26 (0.93–1.74)	0.14		1.18 (0.86–1.62)	0.3		1.32 (0.97–1.81)	0.077		1.32 (0.97–1.81)	0.077
Insomnia	1.60 (1.12–2.30)	0.011		1.13 (0.76–1.67)	0.5		0.98 (0.65–1.47)	>0.9		0.98 (0.65–1.47)	>0.9
Irritable bowel syndrome	1.23 (0.77–1.98)	0.4		0.61 (0.34–1.07)	0.091		0.74 (0.43–1.26)	0.3		0.52 (0.28–0.93)	0.031
Palpitations	1.47 (1.02–2.13)	0.040		0.96 (0.64–1.44)	0.8		0.91 (0.61–1.38)	0.7		0.89 (0.59–1.35)	0.6
Sleep apnea	1.47 (1.23–1.77)	<0.001		1.13 (0.93–1.38)	0.2		1.14 (0.94–1.38)	0.2		1.14 (0.94–1.38)	0.2
Tinnitus	2.56 (1.23–5.82)	0.017		1.33 (0.57–3.26)	0.5		1.33 (0.57–3.26)	0.5		0.89 (0.33–2.32)	0.8

*Baseline was 180–121 days before the index date. The 3-month follow-up periods were nonoverlapping periods after the index date: 0–89 days (0–3 months), 90–179 days (3–6 months), 180–269 days (6–9 months), and 270–365 days (9–12 months). Because of insufficient cell size, prevalence ratios were not calculated for chills, intracranial abscess, meningitis, irritability or anger, general paresis, or hypoactive sexual desire disorder. IACCI, infection-associated chronic conditions and illnesses; PR, prevalence ratio.

### Factors Associated with Period Prevalence of Symptoms

Patients with underlying conditions had higher prevalence of symptoms during baseline and across each follow-up period compared with patients without underlying conditions (Appendix Table 2, Figures 1, 2). Women had higher prevalence of several symptoms (e.g., joint pain and myalgia) than men at baseline and throughout most follow-up periods. Prevalence of erythema nodosum or multiforme, fatigue, and anxiety were similar among women and men at baseline but were higher among women in the periods after index date (Appendix Table 3, Figures 3, 4).

Compared with patients in other age groups, patients >65 years of age had higher prevalence of many symptoms, including weakness, dyspnea, and joint pain, at baseline and during most follow-up periods but had lower prevalence of fever and erythema nodosum or multiforme 0–3 months after index date (Appendix Table 4, Figure 5, 6). Compared with patients who did not receive fluconazole, patients who received fluconazole generally had similar prevalence of symptoms, such as chest pain, dyspnea, fatigue, and fever, at baseline but had higher prevalence during follow-up periods (Appendix Table 5, Figures 7, 8). Approximately 4% of patients who did not receive fluconazole received a >30-day supply of another azole medication (data not shown). Compared with patients with pulmonary or other or unspecified coccidioidomycosis types, patients with extrapulmonary disease had lower prevalence of respiratory symptoms (e.g., cough and dyspnea) and higher prevalence of headache 0–3 months after index date and higher prevalence of weakness, weight loss, fever, and tinnitus 9–12 months after index date (Appendix Table 6, Figures 9, 10).

## Discussion

In this claims-based analysis of patients with commercial health insurance or Medicare, the period prevalence of several coccidioidomycosis-associated symptoms, such as fatigue, joint pain, and weakness, remained significantly elevated for up to 12 months after coccidioidomycosis index date compared with baseline. Most IACCI symptoms were less persistent in patients with coccidioidomycosis, although dizziness and anxiety were marginally elevated at 9–12 months post–index date compared with baseline, and anxiety appeared to increase over time. In addition, the actual duration of many symptoms is likely longer than estimated in our study, because most patients experience substantial delays between symptom onset and coccidioidomycosis index date (*1*,*10*). Our findings corroborate previous data about long-term persistence of symptoms, such as fatigue (*6*), and suggest that the spectrum of long-term symptoms in patients with coccidioidomycosis might be broader and more persistent for some patients than previously recognized (*11*,*12*). Coccidioidomycosis symptoms can be debilitating and can result in lost work and school attendance (*1*,*6*,*7*), making clinical and public health efforts to better characterize and address those symptoms a priority.

Period prevalence of most symptoms was higher in patients with underlying conditions during baseline and most follow-up periods, likely reflecting higher baseline rates of some symptoms and higher rates of severe coccidioidomycosis in this patient population (*4*). Similarly, higher prevalence of symptoms in older patients might reflect differences in baseline health status and the possibility of more severe infections because of higher rates of underlying conditions (*13*). Although male sex is considered a risk factor for susceptibility to *Coccidioides* infection and severe disease (*14*,*15*), we found higher prevalence of several symptoms in women. Higher baseline prevalence of symptoms in women might be attributable to differences in healthcare use or self-reporting of symptoms (*16*,*17*), whereas higher post–index date prevalence of erythema nodosum or multiforme and fatigue in women could also reflect sex-related differences in the immune response to infection (*18*). Patients who received fluconazole generally had higher post–index date prevalence of many symptoms than patients who did not receive fluconazole, as has been observed previously (*3*). That finding likely reflects increased severity of illness in patients who are prescribed antifungal medications for coccidioidomycosis or fluconazole-related side effects (*5*,*19*).

Although the physiologic underpinnings of chronic coccidioidomycosis-related fatigue are not well understood, some studies have pointed to the possible role of diminished aerobic capacity or postinfectious mitochondrial dysfunction (*20*,*21*). Deconditioning could also play a role in chronic fatigue, and referral to physical therapists might be necessary (*21*). Some symptoms, such as persistent joint pain, might be related to patients’ immune response to infection, rather than the infection itself (*22*).

The mechanisms that might explain the marginal post–index date increases in anxiety that we observed are unclear; few studies have investigated the psychological effects of coccidioidomycosis (*11*,*12*). Data from patients with IACCIs point toward illness-related physical and psychological stress, social and economic effects, or immune response dysregulation as possible drivers for postinfection anxiety, which can persist or emerge months after infection (*23*). Further clinical investigations could shed light on the prevalence, persistence, and causes of anxiety or other psychological symptoms in patients with coccidioidomycosis. Future work could also address the effects of persistent coccidioidomycosis symptoms on patient quality of life (*24*).

The first limitation of this study was our reliance on ICD-10-CM codes, which are inherently subject to misclassification and undercoding. Patients were not actively followed, and we relied on diagnosis codes to assess the presence of symptoms during visits; however, providers might not code all associated symptoms during coccidioidomycosis-related visits (*25*). Thus, our symptom prevalence estimates likely underestimate the true prevalence in patients with coccidioidomycosis. Absolute symptom prevalence was substantially higher in a prospective study of 36 patients with pulmonary coccidioidomycosis in which symptom presence was directly evaluated every 4 weeks for 24 weeks (*3*). However, the relative trends appeared similar to those observed in our study, which lend credence to our results. Administrative datasets also offer unique opportunities to study large patient populations, which are difficult to achieve with other sources of fungal disease data, and several studies have similarly assessed coccidioidomycosis symptoms using ICD-10-CM codes (*8*,*9*). Additional large-scale studies with direct patient follow-up or medical chart review could validate our findings by directly measuring symptom prevalence and persistence over longer periods. Second, some symptoms coded in claims data could also have been unrelated to coccidioidomycosis. The substantial increase in symptoms in the months around the index date suggests the observed trends were largely related to coccidioidomycosis, but follow-up case–control studies could help corroborate those findings by comparing symptom trends between patient populations with and without coccidioidomycosis. Third, only patients with commercial health insurance were included in our analysis, meaning our results might not represent the illness experience of those with other insurance types or without health insurance. Fourth, we also lacked data on socioeconomic status and race/ethnicity, which limited our ability to assess potential social determinants of health in symptom prevalence and trends. Finally, the high proportion of patients with unspecified coccidioidomycosis ICD-10-CM codes limited our ability to directly compare symptom trends in patients with pulmonary disease with those of patients with extrapulmonary disease.

In conclusion, our findings provide evidence about the long-term duration of selected symptoms in patients with coccidioidomycosis, which could help clinicians manage patient symptoms long after initial diagnosis and provide information to counsel patients during follow-up evaluation. In addition, these findings can help inform estimates of the overall health and economic burden of coccidioidomycosis to inform related public health interventions and provide additional rationale for ongoing efforts to develop a vaccine against coccidioidomycosis.

Appendix Additional information about persistence of symptoms among commercially insured patients with coccidioidomycosis, United States, 2017–2023
