# Supplementary material for: Persistence of Symptoms among Commercially Insured Patients with Coccidioidomycosis, United States, 2017–2023
Source: Emerg Infect Dis. 2025 Dec;31(Suppl 2):S38–44. doi: 10.3201/eid3114.250022 (PMC12829482; doi:10.3201/eid3114.250022)
Supplement: Appendix — Additional information about persistence of symptoms among commercially insured patients with coccidioidomycosis, United States, 2017–2023 [file 25-0022-Techapp-s1.pdf]

Article DOI: https://doi.org/10.3201/eid3114.250022

EID cannot ensure accessibility for supplementary materials supplied by authors. Readers who have difficulty accessing supplementary content should contact the authors for assistance.

Persistence of Symptoms among Commercially Insured Patients with Coccidioidomycosis, United States, 2017–2023

Appendix

Appendix Table 1. International Classification of Diseases, 10th Revision, Clinical Modification (ICD-10-CM) codes to identify underlying conditions, diagnoses, and conditions of interest in an analysis of U.S. commercial insurance claims data*

Category	Conditions/symptoms	Code(s)
Underlying conditions	Asthma	J45
	Autoimmune inflammatory disease	G35, G70, K50, K51, L40, L93, M02.3, M05, M06, M08, M33, M35.2, M45
	Cancer	C00–C96, excluding C44
	COPD	J43–J44
	Diabetes	E10–E13
	Hypertension	I10
	HIV	B20, Z21
	Solid organ or stem cell transplant or transplant complications	T86, Z94 (excluding Z94.7), Z95.2, Z95.3
Coccidioidomycosis diagnosis codes	Coccidioidomycosis	B38
	Acute pulmonary coccidioidomycosis	B38.0
	Chronic pulmonary coccidioidomycosis	B38.1
	Pulmonary coccidioidomycosis	B38.2
	Cutaneous coccidioidomycosis	B38.3
	Coccidioidomycosis meningitis	B38.4
	Disseminated coccidioidomycosis	B38.7
	Prostatic coccidioidomycosis	B38.81
	Other forms of coccidioidomycosis	B38.89
	Coccidioidomycosis, unspecified	B38.9
Coccidioidomycosis-associated symptoms	Abnormal weight loss	R63.4
	Chest pain	R07.9
	Chills without fever	R68.83
	Cough	R05
	Erythema nodosum or multiforme	L52, L51
	Fatigue or malaise or chronic fatigue syndrome	R53.83, G93.32
	Fever	R50.9
	Generalized hyperhidrosis	R61
	Headache	R51.9
	Myalgia	M79.1
	Pain in joint	M25.50
	Shortness of breath	R06.02
	Weakness	M62.81, R53.1
Select IACCI-associated symptoms	Depression	F32
	Dizziness	R42
	General paresis	A52.17
	Generalized anxiety disorder	F41.1

Category	Conditions/symptoms	Code(s)
	Hypoactive sexual desire disorder	F52.0
	Insomnia	G47.00
	Irritability and anger	R45.4
	Irritable bowel syndrome	K58
	Palpitations	R00.2
	Sleep apnea	G47.30
	Tinnitus	H93.1

*Abbreviations: IACCI = infection associated chronic conditions and illnesses. COPD = chronic obstructive pulmonary disease

Appendix Table 2. Prevalence of symptoms in coccidioidomycosis patients with underlying conditions compared to those without during baseline and four follow-up periods after index date — United States, July 2017–January 2023*

	−6 to −4 mo (Baseline)			0 to 3 mo			3 to 6 mo			6 to 9 mo			9 to 12 mo		
Characteristic	PR	95% CI	p-value	PR	95% CI	p-value	PR	95% CI	p-value	PR	95% CI	p-value	PR	95% CI	p-value
Coccidioidomycosis-associated symptoms															
Weight loss	9.97	1.31, 76.0	0.026	3.77	1.97, 7.18	<0.001	1.99	0.71, 5.58	0.2	–	–	–	6.14	1.41, 26.6	0.015
Chest pain	1.81	1.24, 2.65	0.002	1.71	1.39, 2.12	<0.001	1.98	1.32, 2.98	0.001	1.38	0.89, 2.14	0.2	1.56	1.03, 2.35	0.034
Cough	2.36	1.79, 3.12	<0.001	1.39	1.23, 1.56	<0.001	1.41	1.13, 1.78	0.003	1.57	1.20, 2.05	0.001	1.32	1.02, 1.71	0.033
Dyspnea	3.38	2.24, 5.10	<0.001	2.20	1.85, 2.62	<0.001	1.90	1.40, 2.59	<0.001	1.92	1.37, 2.68	<0.001	2.30	1.56, 3.39	<0.001
EN/EM	–	–	–	0.88	0.53, 1.47	0.6	1.34	0.39, 4.57	0.6	3.07	0.34, 27.4	0.3	–	–	–
Fatigue	1.65	1.17, 2.33	0.004	1.24	1.04, 1.47	0.016	1.46	1.09, 1.96	0.011	1.36	1.02, 1.82	0.034	1.74	1.28, 2.36	<0.001
Fever	1.31	0.83, 2.08	0.2	1.21	0.98, 1.50	0.082	1.37	0.84, 2.25	0.2	1.29	0.74, 2.27	0.4	1.85	1.06, 3.24	0.031
Hyperhidrosis	1.02	0.23, 4.56	>0.9	0.95	0.50, 1.79	0.9	0.64	0.20, 2.09	0.5	1.02	0.23, 4.56	>0.9	0.58	0.13, 2.57	0.5
Headache	0.77	0.33, 1.76	0.5	0.99	0.64, 1.55	>0.9	0.92	0.47, 1.82	0.8	1.36	0.60, 3.07	0.5	1.04	0.56, 1.93	>0.9
Myalgia	1.07	0.34, 3.37	>0.9	0.66	0.36, 1.22	0.2	1.15	0.41, 3.22	0.8	2.30	0.62, 8.48	0.2	3.84	0.45, 32.8	0.2
Pain in joint	1.63	1.27, 2.09	<0.001	1.43	1.22, 1.68	<0.001	1.49	1.19, 1.86	<0.001	1.33	1.06, 1.65	0.012	1.14	0.92, 1.40	0.2
Weakness	2.24	1.20, 4.19	0.011	2.30	1.65, 3.21	<0.001	3.40	1.91, 6.04	<0.001	3.40	1.99, 5.82	<0.001	1.66	1.09, 2.54	0.019
IACCI-associated symptoms															
Depression	2.41	1.48, 3.92	<0.001	2.16	1.61, 2.89	<0.001	2.63	1.67, 4.17	<0.001	1.78	1.18, 2.70	0.006	1.79	1.18, 2.73	0.007
Dizziness	1.92	1.04, 3.55	0.038	1.87	1.29, 2.70	<0.001	1.12	0.65, 1.91	0.7	1.24	0.69, 2.21	0.5	1.37	0.84, 2.25	0.2
Anxiety	1.03	0.64, 1.66	0.9	1.20	0.87, 1.65	0.3	1.35	0.86, 2.11	0.2	1.05	0.70, 1.58	0.8	1.99	1.27, 3.14	0.003
Insomnia	1.64	0.89, 3.01	0.11	1.57	1.08, 2.29	0.018	3.75	1.84, 7.65	<0.001	1.31	0.72, 2.37	0.4	3.15	1.53, 6.51	0.002
IBS	1.61	0.76, 3.41	0.2	1.75	1.05, 2.92	0.031	2.88	0.96, 8.64	0.060	2.17	0.86, 5.49	0.10	3.32	0.95, 11.6	0.060
Palpitations	1.81	0.97, 3.36	0.061	1.78	1.20, 2.63	0.004	1.26	0.69, 2.30	0.4	1.77	0.93, 3.38	0.083	1.13	0.61, 2.08	0.7
Sleep apnea	2.11	1.53, 2.91	<0.001	2.13	1.73, 2.60	<0.001	2.19	1.61, 2.96	<0.001	1.94	1.45, 2.61	<0.001	2.26	1.66, 3.06	<0.001
Tinnitus	0.61	0.17, 2.28	0.5	0.87	0.44, 1.74	0.7	2.30	0.62, 8.48	0.2	0.55	0.17, 1.72	0.3	1.28	0.31, 5.34	0.7

*Baseline was 180 to 121 d before index date. Three-month follow-up periods were non-overlapping periods post-index date: 0–89 d (0–3 mo), 90–179 d (3–6 mo), 180–69 d (6–9 mo), and 270–365 d (9–12 mo). Abbreviations: PR = prevalence ratio; IACCI = infection associated chronic conditions and illnesses; EN/EM = Erythema nodosum or multiforme; IBS = irritable bowel syndrome. Due to insufficient cell size, prevalence ratios were not calculated for chills, irritability or anger, general paresis, or hypoactive sexual desire disorder

Appendix Table 3. Prevalence of symptoms among female versus male patients with coccidioidomycosis during baseline and four follow-up periods after index date — United States, July 2017–January 2023*

Characteristic	−6 to −4 mo (Baseline)			0 to 3 mo			3 to 6 mo			6 to 9 mo			9 to 12 mo		
	PR	95% CI	p-value	PR	95% CI	p-value	PR	95% CI	p-value	PR	95% CI	p-value	PR	95% CI	p-value
Coccidioidomycosis-associated symptoms															
Weight loss	1.92	0.65, 5.72	0.2	0.75	0.43, 1.30	0.3	0.85	0.34, 2.16	0.7	3.47	1.13, 10.6	0.029	0.85	0.34, 2.16	0.7
Chest pain	1.46	1.03, 2.08	0.034	1.08	0.86, 1.37	0.5	1.35	0.94, 1.95	0.11	1.17	0.77, 1.79	0.5	1.60	1.08, 2.37	0.019
Cough	1.26	1.00, 1.60	0.053	1.23	1.09, 1.40	0.001	1.43	1.15, 1.78	0.001	1.21	0.94, 1.56	0.14	1.48	1.15, 1.90	0.002
Dyspnea	1.22	0.89, 1.68	0.2	1.10	0.93, 1.30	0.3	1.38	1.04, 1.82	0.023	1.14	0.84, 1.53	0.4	1.59	1.13, 2.23	0.007
EN/EM	61,048,103	0.00, Inf	>0.9	2.08	1.20, 3.59	0.009	1.28	0.39, 4.19	0.7	0.71	0.12, 4.25	0.7	0.53	0.05, 5.88	0.6
Fatigue	1.13	0.82, 1.56	0.5	1.24	1.02, 1.50	0.032	1.41	1.07, 1.87	0.015	1.13	0.85, 1.48	0.4	1.47	1.11, 1.95	0.008
Fever	0.78	0.50, 1.22	0.3	0.78	0.61, 0.99	0.040	1.40	0.87, 2.25	0.2	1.20	0.70, 2.07	0.5	1.23	0.74, 2.04	0.4
Hyperhidrosis	2.67	0.52, 13.7	0.2	1.00	0.50, 2.02	>0.9	0.89	0.27, 2.91	0.8	6.40	0.77, 53.1	0.085	2.67	0.52, 13.7	0.2
Headache	1.54	0.66, 3.59	0.3	1.32	0.79, 2.23	0.3	1.87	0.92, 3.78	0.082	2.27	0.98, 5.24	0.055	1.98	1.04, 3.78	0.038
Myalgia	5.34	1.17, 24.3	0.030	2.03	0.95, 4.34	0.069	2.13	0.73, 6.23	0.2	1.49	0.48, 4.70	0.5	5.34	0.62, 45.6	0.13
Pain in joint	1.52	1.20, 1.92	<0.001	1.09	0.89, 1.34	0.4	1.45	1.17, 1.79	<0.001	1.38	1.12, 1.71	0.003	1.25	1.02, 1.53	0.035
Weakness	1.41	0.81, 2.44	0.2	1.19	0.85, 1.67	0.3	1.64	1.04, 2.58	0.033	1.14	0.76, 1.73	0.5	1.24	0.83, 1.84	0.3
IACCI-associated symptoms															
Depression	1.93	1.25, 2.97	0.003	1.88	1.34, 2.64	<0.001	3.12	2.01, 4.84	<0.001	2.72	1.78, 4.16	<0.001	2.49	1.63, 3.79	<0.001
Dizziness	1.69	0.95, 2.98	0.072	0.95	0.62, 1.44	0.8	2.53	1.42, 4.52	0.002	1.57	0.88, 2.80	0.12	1.68	1.04, 2.73	0.036
Anxiety	1.62	1.00, 2.62	0.049	1.71	1.12, 2.63	0.014	2.22	1.40, 3.52	<0.001	1.93	1.27, 2.96	0.002	2.03	1.32, 3.12	0.001
Insomnia	0.86	0.49, 1.52	0.6	1.10	0.70, 1.71	0.7	1.39	0.81, 2.38	0.2	3.02	1.57, 5.81	<0.001	1.82	1.01, 3.30	0.048
IBS	2.24	1.06, 4.74	0.035	2.99	1.46, 6.13	0.003	1.83	0.72, 4.63	0.2	3.02	1.20, 7.65	0.019	7.47	1.70, 32.8	0.008
Palpitations	1.32	0.75, 2.34	0.3	1.39	0.87, 2.22	0.2	2.36	1.26, 4.42	0.007	1.48	0.81, 2.70	0.2	1.57	0.85, 2.89	0.15
Sleep apnea	0.52	0.38, 0.70	<0.001	0.63	0.49, 0.79	<0.001	0.66	0.50, 0.87	0.003	0.54	0.41, 0.72	<0.001	0.58	0.44, 0.76	<0.001
Tinnitus	1.33	0.36, 4.96	0.7	0.98	0.43, 2.21	>0.9	0.76	0.24, 2.40	0.6	1.07	0.35, 3.30	>0.9	0.64	0.15, 2.67	0.5

*Baseline was 180 to 121 d before index date. Three-month follow-up periods were non-overlapping periods post-index date: 0–89 d (0–3 mo), 90–179 d (3–6 mo), 180–69 d (6–9 mo), and 270–365 d (9–12 mo). Abbreviations: PR = prevalence ratio; IACCI = infection associated chronic conditions and illnesses; EN/EM = Erythema nodosum or multiforme; IBS = irritable bowel syndrome. Due to insufficient cell size, prevalence ratios were not calculated for chills, irritability or anger, general paresis, or hypoactive sexual desire disorder.

Appendix Table 4. Prevalence of symptoms among patients ≥65 compared to those <65 during baseline and four follow-up periods after index date — United States, July 2017–January 2023

Characteristic	−6 to −4 mo (Baseline)			0 to 3 mo			3 to 6 mo			6 to 9 mo			9 to 12 mo		
	PR	95% CI	p-value	PR	95% CI	p-value	PR	95% CI	p-value	PR	95% CI	p-value	PR	95% CI	p-value
Coccidioidomycosis-associated symptoms															
Weight loss	2.32	0.73, 7.37	0.2	1.79	0.94, 3.38	0.075	1.16	0.34, 3.99	0.8	2.42	0.86, 6.83	0.10	4.64	1.84, 11.7	0.001
Chest pain	1.59	1.04, 2.42	0.031	0.87	0.61, 1.23	0.4	1.35	0.85, 2.15	0.2	1.37	0.80, 2.33	0.3	1.19	0.71, 1.98	0.5
Cough	1.76	1.34, 2.32	<0.001	0.87	0.72, 1.05	0.2	1.36	1.03, 1.78	0.028	1.35	0.99, 1.86	0.062	1.27	0.92, 1.74	0.15
Dyspnea	2.19	1.55, 3.10	<0.001	1.49	1.21, 1.82	<0.001	2.16	1.60, 2.93	<0.001	2.05	1.47, 2.86	<0.001	2.09	1.44, 3.04	<0.001
EN/EM	–	–	–	0.21	0.05, 0.88	0.032	1.29	0.28, 5.95	0.7	–	–	–	–	–	–
Fatigue	1.06	0.68, 1.65	0.8	0.89	0.67, 1.19	0.4	0.90	0.60, 1.35	0.6	1.01	0.69, 1.49	>0.9	1.47	1.04, 2.08	0.028
Fever	0.78	0.39, 1.55	0.5	0.59	0.39, 0.89	0.013	1.27	0.68, 2.34	0.5	0.77	0.33, 1.80	0.6	0.93	0.44, 1.94	0.8
Hyperhidrosis	–	–	–	0.62	0.19, 2.03	0.4	0.58	0.07, 4.52	0.6	2.32	0.45, 11.9	0.3	0.97	0.12, 8.01	>0.9
Headache	1.29	0.44, 3.79	0.6	0.57	0.23, 1.42	0.2	0.37	0.09, 1.56	0.2	0.79	0.24, 2.63	0.7	0.31	0.07, 1.26	0.10
Myalgia	1.16	0.26, 5.28	0.8	0.93	0.33, 2.65	0.9	0.89	0.20, 3.94	0.9	0.53	0.07, 4.07	0.5	1.16	0.14, 9.91	0.9
Pain in joint	1.71	1.31, 2.24	<0.001	1.31	1.01, 1.69	0.041	1.70	1.33, 2.17	<0.001	1.34	1.03, 1.76	0.030	1.52	1.19, 1.94	<0.001
Weakness	3.45	1.97, 6.02	<0.001	4.05	2.90, 5.65	<0.001	4.70	3.03, 7.29	<0.001	3.05	1.99, 4.69	<0.001	3.39	2.26, 5.06	<0.001
IACCI-associated symptoms															

Characteristic	−6 to −4 mo (Baseline) PR	95% CI	p-value	0 to 3 mo PR	95% CI	p-value	3 to 6 mo PR	95% CI	p-value	6 to 9 mo PR	95% CI	p-value	9 to 12 mo PR	95% CI	p-value
Depression	1.73	1.06, 2.82	0.027	1.48	0.99, 2.21	0.056	1.69	1.08, 2.65	0.023	1.31	0.81, 2.13	0.3	1.64	1.03, 2.60	0.036
Dizziness	4.73	2.72, 8.22	<0.001	1.79	1.09, 2.91	0.020	2.03	1.12, 3.70	0.020	1.57	0.79, 3.13	0.2	2.84	1.72, 4.67	<0.001
Anxiety	0.77	0.37, 1.61	0.5	1.13	0.64, 1.98	0.7	0.74	0.37, 1.46	0.4	1.16	0.67, 2.00	0.6	0.89	0.49, 1.62	0.7
Insomnia	1.77	0.91, 3.45	0.092	1.57	0.92, 2.71	0.10	1.19	0.58, 2.41	0.6	2.05	1.07, 3.92	0.030	2.29	1.21, 4.30	0.010
IBS	1.39	0.58, 3.37	0.5	1.31	0.58, 2.96	0.5	2.68	1.02, 7.01	0.045	1.61	0.60, 4.32	0.3	1.34	0.38, 4.68	0.6
Palpitations	1.02	0.46, 2.25	>0.9	1.35	0.74, 2.44	0.3	1.66	0.83, 3.32	0.2	1.54	0.74, 3.18	0.2	1.16	0.52, 2.59	0.7
Sleep apnea	2.23	1.64, 3.04	<0.001	1.85	1.43, 2.40	<0.001	2.14	1.60, 2.86	<0.001	2.35	1.77, 3.11	<0.001	2.08	1.55, 2.78	<0.001
Tinnitus	1.66	0.35, 7.95	0.5	–	–	–	2.90	0.88, 9.59	0.081	2.90	0.88, 9.59	0.081	0.83	0.10, 6.72	0.9

*Baseline was 180 to 121 d before index date. Three-month follow-up periods were non-overlapping periods post-index date: 0–89 d (0–3 mo), 90–179 d (3–6 mo), 180–69 d (6–9 mo), and 270–365 d (9–12 mo). Abbreviations: PR = prevalence ratio; IACCI = infection associated chronic conditions and illnesses; EN/EM = Erythema nodosum or multiforme; IBS = irritable bowel syndrome. Due to insufficient cell size, prevalence ratios were not calculated for chills, irritability or anger, general paresis, or hypoactive sexual desire disorder.

Appendix Table 5. Prevalence of symptoms among patients who received fluconazole compared to those who did not during baseline and four follow-up periods after index date — United States, July 2017–January 2023*

Characteristic	−6 to −4 mo (Baseline) PR	95% CI	p-value	0 to 3 mo PR	95% CI	p-value	3 to 6 mo PR	95% CI	p-value	6 to 9 mo PR	95% CI	p-value	9 to 12 mo PR	95% CI	p-value
Coccidioidomycosis-associated symptoms															
Weight loss	0.92	0.32, 2.62	0.9	1.69	0.95, 2.98	0.072	1.84	0.69, 4.89	0.2	1.04	0.40, 2.67	>0.9	1.15	0.46, 2.90	0.8
Chest pain	1.03	0.73, 1.46	0.9	1.59	1.25, 2.03	<0.001	1.30	0.90, 1.88	0.2	1.11	0.73, 1.70	0.6	1.44	0.97, 2.13	0.071
Cough	1.33	1.04, 1.69	0.022	1.55	1.35, 1.76	<0.001	1.57	1.26, 1.97	<0.001	1.21	0.94, 1.56	0.15	1.45	1.12, 1.87	0.004
Dyspnea	0.97	0.71, 1.33	0.9	1.44	1.21, 1.71	<0.001	1.59	1.19, 2.11	0.002	1.08	0.80, 1.46	0.6	1.17	0.84, 1.64	0.3
EN/EM	–	–	–	4.23	2.14, 8.35	<0.001	9.20	1.18, 71.8	0.034	1.38	0.23, 8.25	0.7	–	–	–
Fatigue	0.78	0.56, 1.07	0.12	1.34	1.10, 1.64	0.003	1.56	1.17, 2.08	0.002	1.31	0.99, 1.73	0.059	1.52	1.14, 2.03	0.005
Fever	1.41	0.90, 2.22	0.14	2.09	1.62, 2.71	<0.001	1.21	0.75, 1.94	0.4	0.96	0.56, 1.65	0.9	1.89	1.10, 3.25	0.022
Hyperhidrosis	0.00	0.00, Inf	>0.9	1.93	0.91, 4.09	0.085	1.10	0.34, 3.61	0.9	0.69	0.15, 3.08	0.6	2.30	0.45, 11.8	0.3
Headache	0.77	0.33, 1.77	0.5	1.66	0.96, 2.85	0.068	1.42	0.71, 2.83	0.3	1.96	0.85, 4.51	0.12	2.76	1.35, 5.62	0.005
Myalgia	0.66	0.21, 2.07	0.5	0.75	0.36, 1.55	0.4	1.05	0.38, 2.89	>0.9	0.31	0.08, 1.13	0.076	1.84	0.34, 10.0	0.5
Pain in joint	1.03	0.82, 1.30	0.8	0.88	0.72, 1.07	0.2	0.91	0.74, 1.13	0.4	0.94	0.76, 1.16	0.6	1.04	0.84, 1.27	0.7
Weakness	0.55	0.31, 0.96	0.035	0.99	0.71, 1.39	>0.9	0.92	0.59, 1.43	0.7	0.86	0.57, 1.30	0.5	1.16	0.78, 1.73	0.5
IACCI-associated symptoms															
Depression	0.99	0.65, 1.49	>0.9	1.10	0.79, 1.52	0.6	1.17	0.79, 1.71	0.4	1.10	0.75, 1.60	0.6	0.85	0.58, 1.25	0.4
Dizziness	0.88	0.51, 1.54	0.7	1.38	0.90, 2.12	0.14	1.24	0.73, 2.12	0.4	0.68	0.38, 1.21	0.2	1.28	0.79, 2.07	0.3
Anxiety	1.10	0.69, 1.76	0.7	1.01	0.67, 1.53	>0.9	1.07	0.69, 1.65	0.8	0.84	0.56, 1.26	0.4	0.59	0.39, 0.89	0.012
Insomnia	0.52	0.29, 0.94	0.030	1.05	0.67, 1.64	0.8	0.89	0.52, 1.51	0.7	0.92	0.52, 1.63	0.8	0.84	0.48, 1.50	0.6
IBS	1.12	0.55, 2.26	0.8	0.83	0.44, 1.56	0.6	1.02	0.42, 2.51	>0.9	0.84	0.37, 1.90	0.7	1.53	0.56, 4.21	0.4
Palpitations	1.05	0.59, 1.84	0.9	1.20	0.75, 1.91	0.5	1.26	0.70, 2.26	0.4	0.88	0.49, 1.59	0.7	1.23	0.67, 2.25	0.5
Sleep apnea	0.86	0.65, 1.14	0.3	0.89	0.71, 1.11	0.3	0.82	0.63, 1.06	0.14	0.89	0.69, 1.16	0.4	0.89	0.69, 1.16	0.4
Tinnitus	1.15	0.31, 4.27	0.8	3.31	1.23, 8.89	0.017	4.60	1.01, 21.0	0.049	0.92	0.30, 2.85	0.9	6.44	0.79, 52.3	0.081

*Fluconazole receipt was defined as receipt of a ≥30-d supply. Baseline was 180 to 121 d before index date. Three-month follow-up periods were non-overlapping periods post-index date: 0–89 d (0–3 mo), 90–179 d (3–6 mo), 180–69 d (6–9 mo), and 270–365 d (9–12 mo). Abbreviations: PR = prevalence ratio; IACCI = infection associated chronic conditions and illnesses; EN/EM = Erythema nodosum or multiforme; IBS = irritable bowel syndrome. Due to insufficient cell size, prevalence ratios were not calculated for chills, irritability or anger, general paresis, or hypoactive sexual desire disorder.

Appendix Table 6. Prevalence of symptoms among patients with extrapulmonary coccidioidomycosis compared to pulmonary or other/unspecified coccidioidomycosis during baseline and four follow-up periods after index date — United States, July 2017–January 2023*

	−6 to −4 mo (Baseline)			0 to 3 mo			3 to 6 mo			6 to 9 mo			9 to 12 mo		
Characteristic	PR	95% CI	p-value	PR	95% CI	p-value	PR	95% CI	p-value	PR	95% CI	p-value	PR	95% CI	p-value
Coccidioidomycosis-associated symptoms															
Weight loss	1.28	0.17, 9.70	0.8	0.68	0.17, 2.76	0.6	3.32	0.97, 11.3	0.056	2.21	0.51, 9.59	0.3	4.74	1.58, 14.2	0.005
Chest pain	0.42	0.14, 1.31	0.14	0.68	0.37, 1.25	0.2	1.29	0.64, 2.60	0.5	1.05	0.43, 2.56	>0.9	1.44	0.71, 2.92	0.3
Cough	0.48	0.23, 1.00	0.050	0.36	0.22, 0.59	<0.001	0.78	0.46, 1.33	0.4	0.54	0.26, 1.13	0.10	1.00	0.59, 1.71	>0.9
Dyspnea	0.35	0.11, 1.08	0.068	0.54	0.32, 0.89	0.016	0.64	0.31, 1.34	0.2	0.64	0.29, 1.43	0.3	0.93	0.44, 1.95	0.8
EN/EM	–	–	–	0.94	0.30, 2.97	>0.9	1.66	0.21, 12.9	0.6	4.15	0.47, 36.9	0.2	–	–	–
Fatigue	0.73	0.33, 1.63	0.4	0.84	0.53, 1.33	0.5	0.36	0.14, 0.97	0.043	1.03	0.57, 1.84	>0.9	1.16	0.66, 2.04	0.6
Fever	1.17	0.48, 2.85	0.7	1.29	0.82, 2.03	0.3	1.63	0.72, 3.72	0.2	1.41	0.52, 3.87	0.5	3.89	2.06, 7.33	<0.001
Hyperhidrosis	2.77	0.34, 22.8	0.3	0.55	0.08, 4.02	0.6	–	–	–	–	–	–	2.77	0.34, 22.8	0.3
Headache	1.66	0.39, 7.04	0.5	3.61	1.86, 7.01	<0.001	1.66	0.51, 5.38	0.4	2.26	0.69, 7.48	0.2	2.37	0.94, 5.97	0.067
Myalgia	–	–	–	0.59	0.08, 4.32	0.6	2.55	0.58, 11.2	0.2	–	–	–	–	–	–
Pain in joint	1.02	0.62, 1.67	>0.9	0.79	0.48, 1.28	0.3	0.91	0.56, 1.46	0.7	1.17	0.77, 1.79	0.5	1.10	0.72, 1.67	0.7
Weakness	1.41	0.52, 3.87	0.5	1.39	0.75, 2.60	0.3	1.17	0.48, 2.85	0.7	1.68	0.83, 3.41	0.2	3.62	2.20, 5.95	<0.001
IACCI-associated symptoms															
Depression	0.59	0.19, 1.85	0.4	1.02	0.51, 2.05	>0.9	1.22	0.58, 2.59	0.6	1.78	0.95, 3.36	0.072	1.44	0.71, 2.92	0.3
Dizziness	1.89	0.76, 4.69	0.2	1.72	0.85, 3.51	0.13	1.33	0.49, 3.63	0.6	0.36	0.05, 2.59	0.3	1.34	0.55, 3.28	0.5
Anxiety	0.77	0.24, 2.41	0.6	0.60	0.19, 1.88	0.4	1.11	0.45, 2.70	0.8	0.98	0.40, 2.37	>0.9	0.98	0.40, 2.37	>0.9
Insomnia	0.74	0.18, 3.01	0.7	1.71	0.80, 3.66	0.2	1.00	0.31, 3.16	>0.9	1.16	0.36, 3.69	0.8	0.37	0.05, 2.65	0.3
IBS	1.14	0.28, 4.75	0.9	0.92	0.22, 3.79	>0.9	0.00	0.00, Inf	>0.9	2.49	0.75, 8.29	0.14	0.00	0.00, Inf	>0.9
Palpitations	1.13	0.36, 3.60	0.8	0.50	0.12, 2.00	0.3	0.77	0.19, 3.16	0.7	0.81	0.20, 3.32	0.8	0.83	0.20, 3.40	0.8
Sleep apnea	0.98	0.53, 1.81	>0.9	0.79	0.45, 1.37	0.4	0.59	0.28, 1.23	0.2	0.59	0.28, 1.22	0.2	0.59	0.28, 1.22	0.2
Tinnitus	–	–	–	1.58	0.37, 6.68	0.5	1.51	0.20, 11.6	0.7	3.32	0.73, 15.0	0.12	5.53	1.13, 27.2	0.035

*Baseline was 180 to 121 d before index date. Three-month follow-up periods were non-overlapping periods post-index date: 0–89 d (0–3 mo), 90–179 d (3–6 mo), 180–69 d (6–9 mo), and 270–365 d (9–12 mo). Abbreviations: PR = prevalence ratio; IACCI = infection associated chronic conditions and illnesses; EN/EM = Erythema nodosum or multiforme; IBS = irritable bowel syndrome. Due to insufficient cell size, prevalence ratios were not calculated for chills, irritability or anger, general paresis, or hypoactive sexual desire disorder.

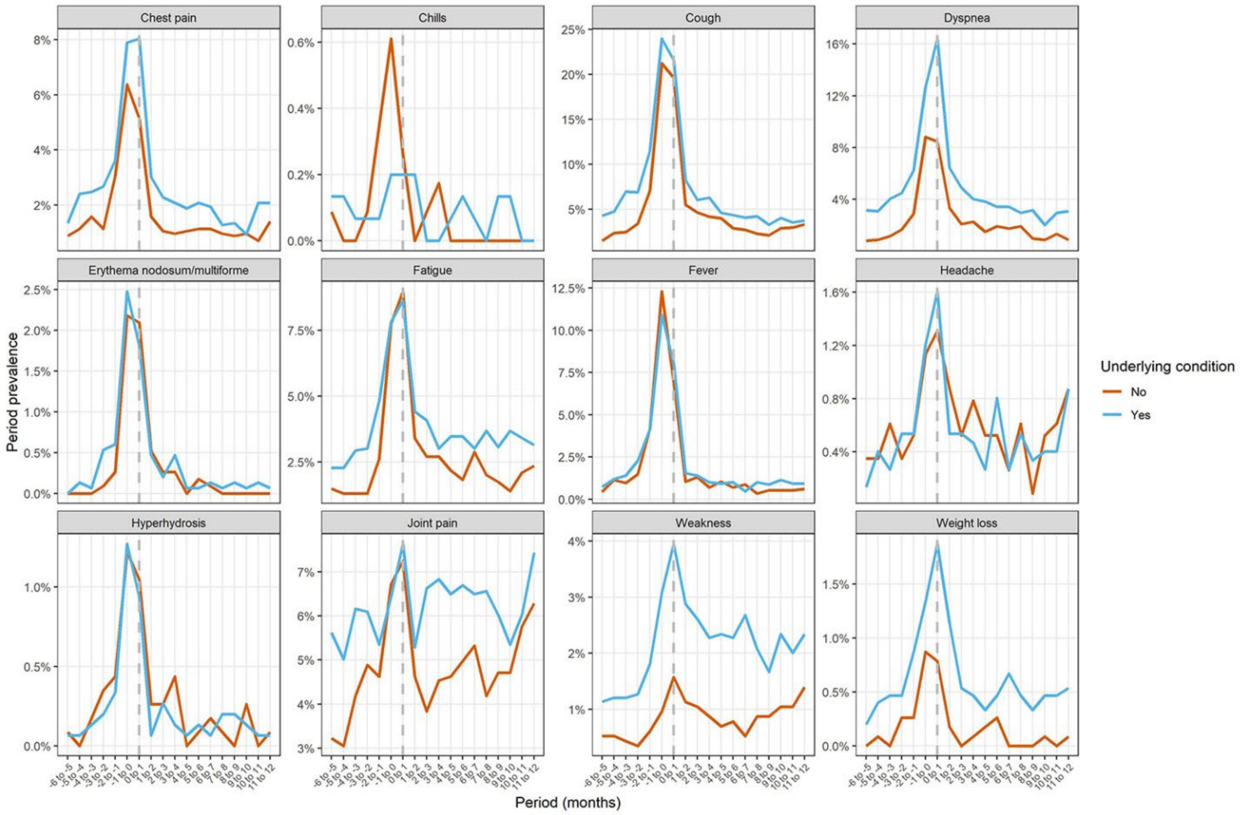

Appendix Figure 1. Period prevalence of selected coccidioidomycosis-associated symptoms among patients with coccidioidomycosis by underlying condition — United States, July 2017–January 2023. The index period (0–29 days after index date) is shown with a dotted line. Underlying conditions included asthma or chronic obstructive pulmonary disease, diabetes, and immunosuppression. Data for myalgia not shown due to period prevalence <1%.

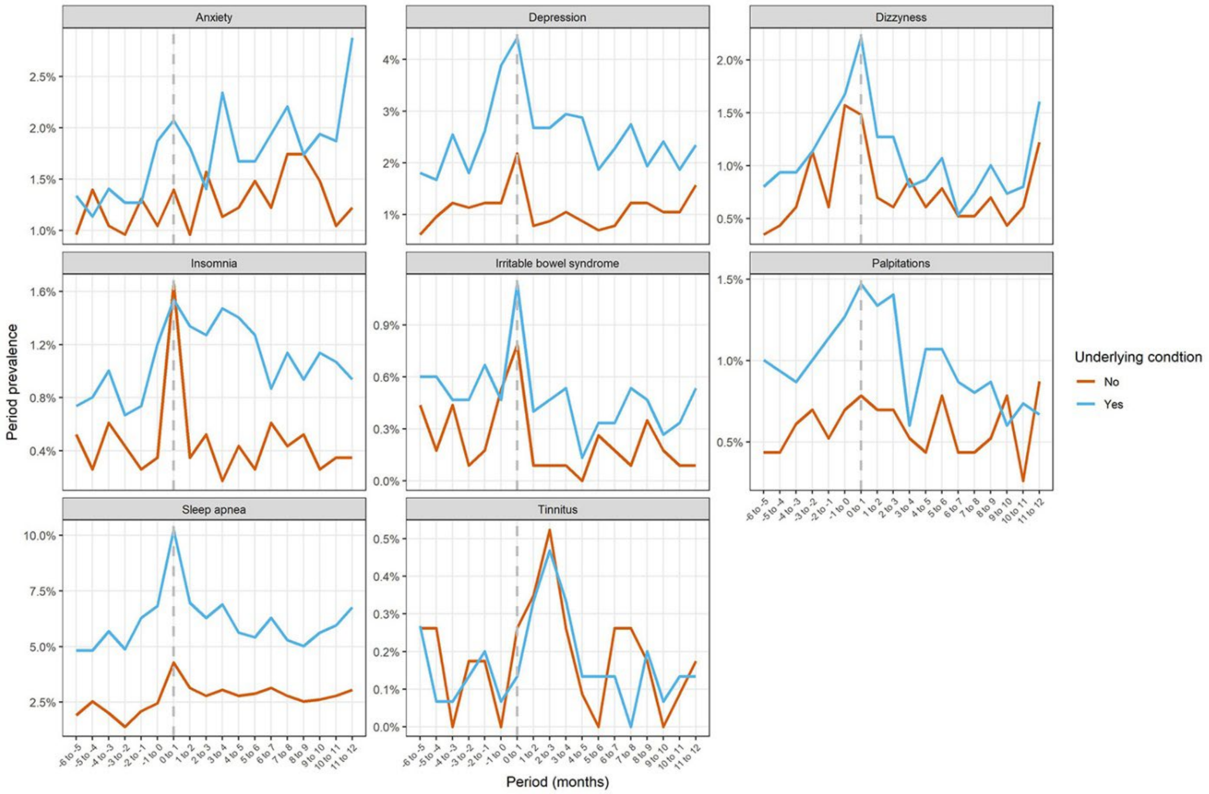

Appendix Figure 2. Period prevalence of selected IACCI-associated symptoms among patients with coccidioidomycosis by underlying condition — United States, July 2017–January 2023. The index period (0–29 days after index date) is shown with a dotted line. Underlying conditions included asthma or chronic obstructive pulmonary disease, diabetes, and immunosuppression. Abbreviations: IACCI = infection-associated chronic conditions and illnesses. Anger, hypoactive sexual desire disorder, and paresis are not shown because prevalence was <0.1% in all periods.

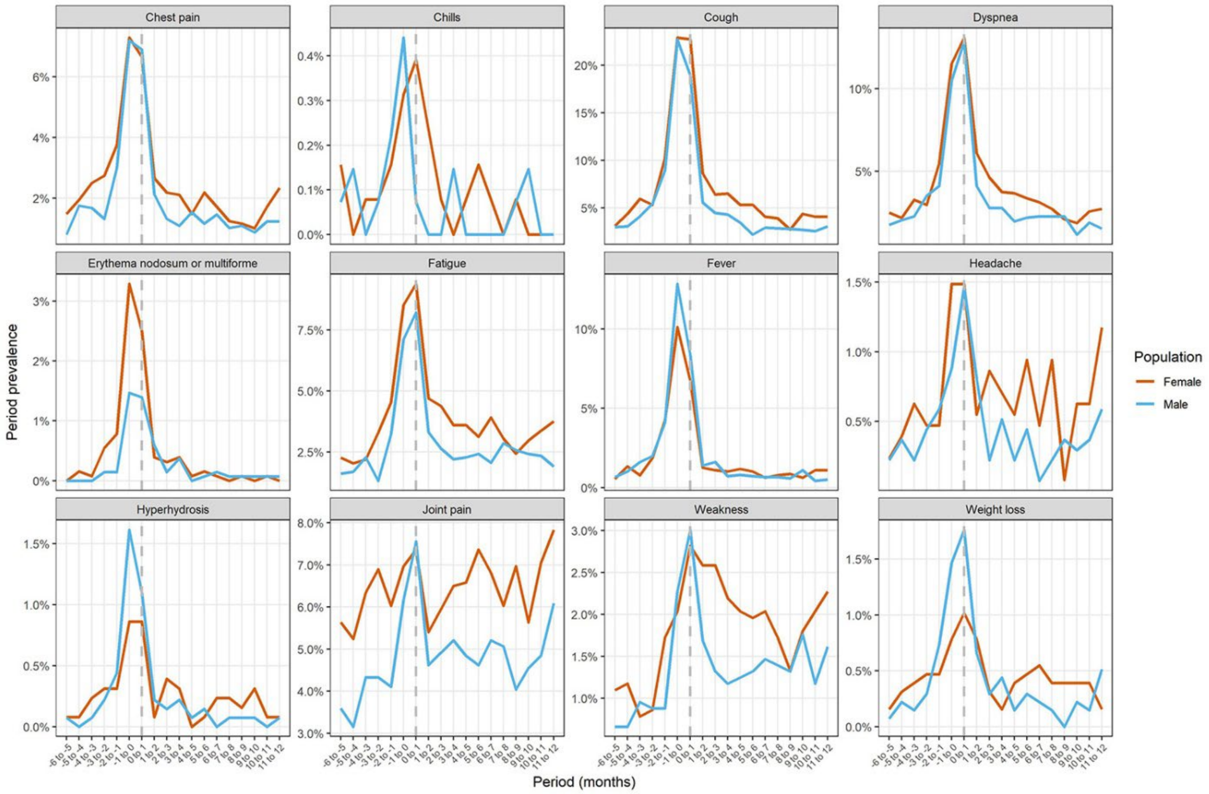

Appendix Figure 3. Period prevalence of selected coccidioidomycosis-associated symptoms among patients with coccidioidomycosis by sex — United States, July 2017–January 2023. The index period (0–29 days after index date) is shown with a dotted line. Data for myalgia not shown due to prevalence <1%.

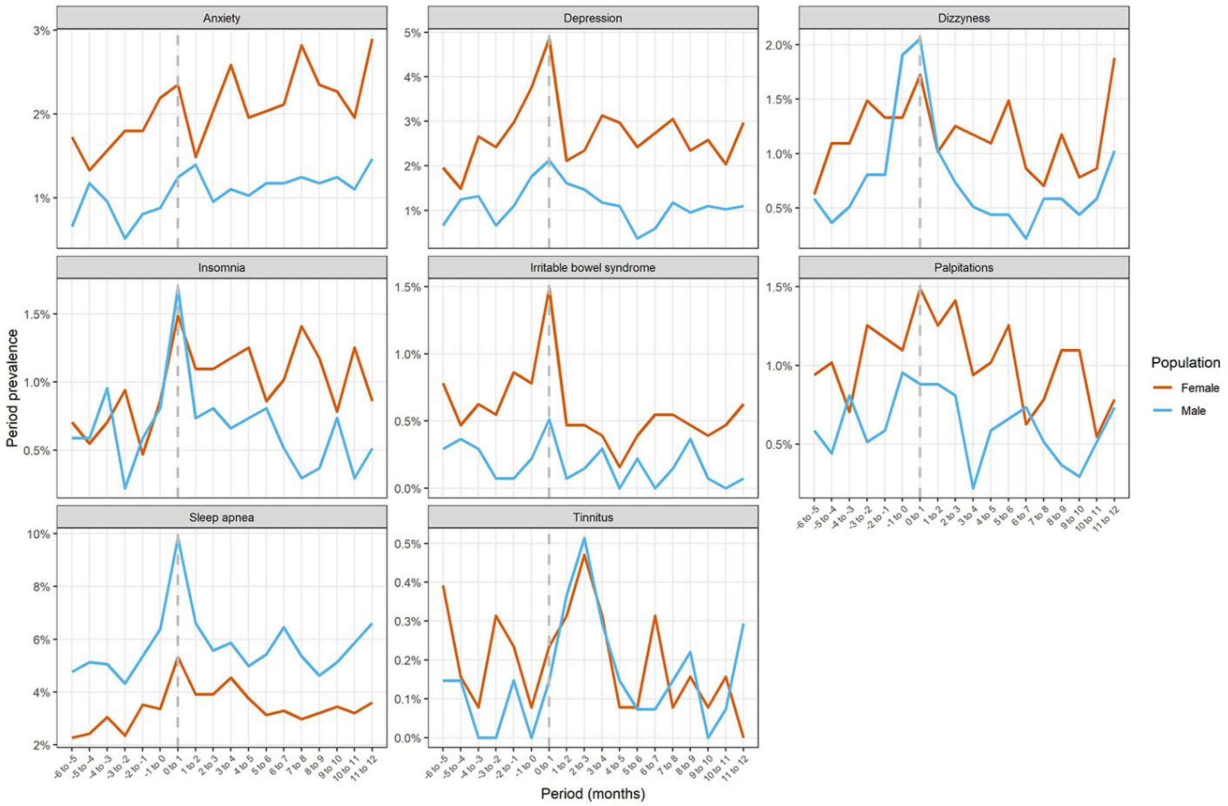

Appendix Figure 4. Period prevalence of selected IACCI-associated symptoms among patients with coccidioidomycosis by sex— United States, July 2017–January 2023. The index period (0–29 days after index date) is shown with a dotted line. Abbreviations: IACCI = infection-associated chronic conditions and illnesses. Anger, hypoactive sexual desire disorder, and paresis are not shown because prevalence was <0.1% in all periods.

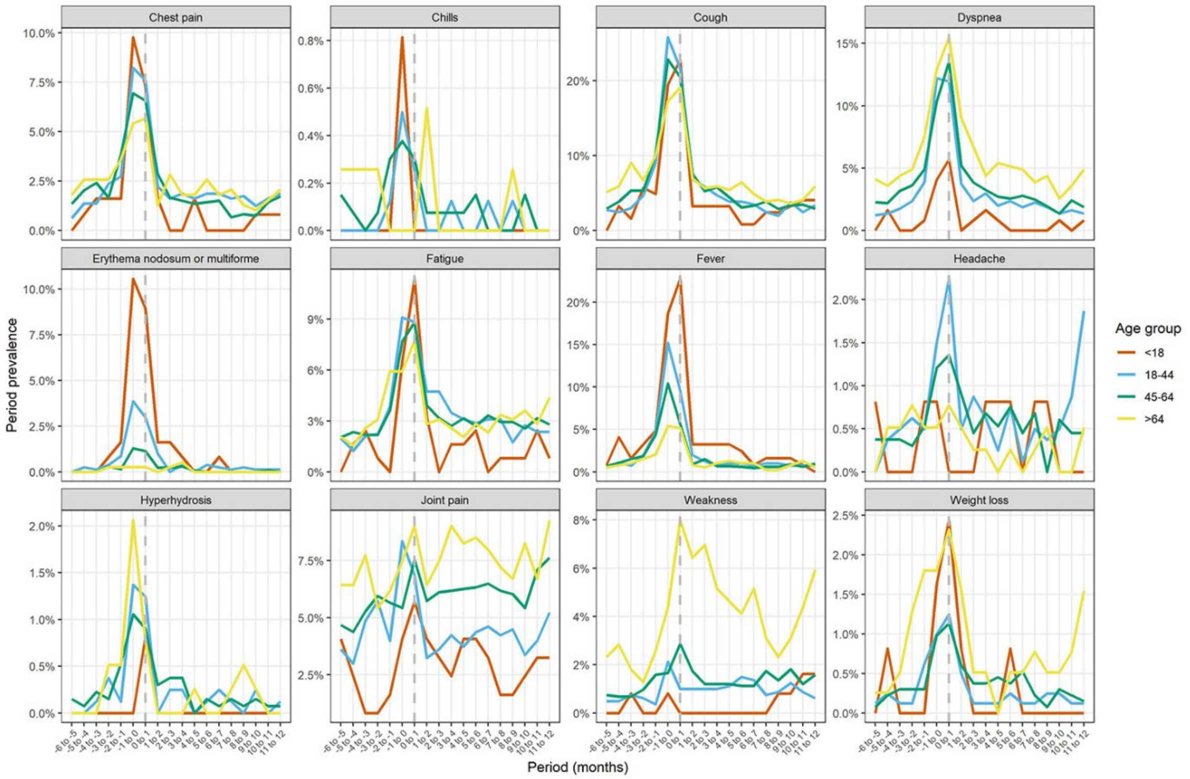

Appendix Figure 5. Period prevalence of selected coccidioidomycosis-associated symptoms among patients with coccidioidomycosis by age group — United States, July 2017–January 2023. The index period (0–29 days after index date) is shown with a dotted line. Data for myalgia not shown due to prevalence <1%.

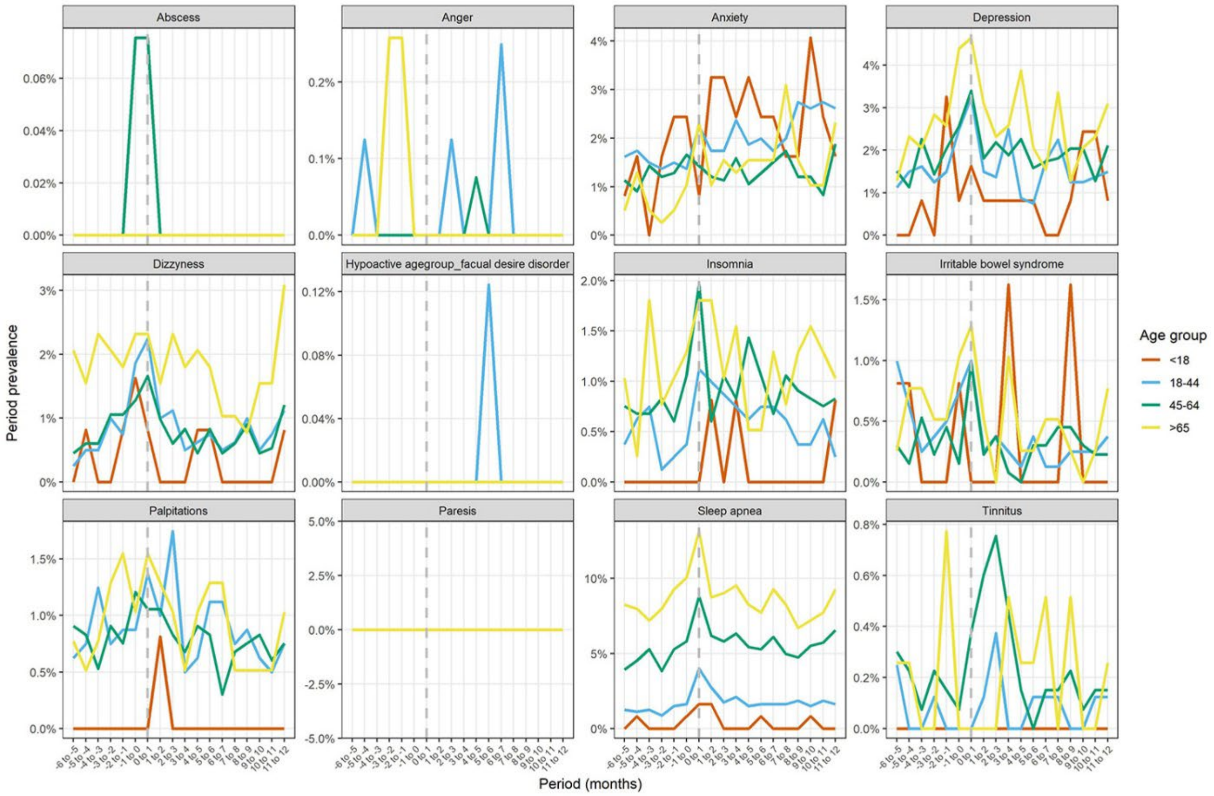

Appendix Figure 6. Period prevalence of selected IACCI-associated symptoms among patients with coccidioidomycosis by age group — United States, July 2017–January 2023. The index period (0–29 days after index date) is shown with a dotted line. Abbreviations: IACCI = infection-associated chronic conditions and illnesses. Anger, hypoactive sexual desire disorder, and paresis are not shown because prevalence was <0.1% in all periods.

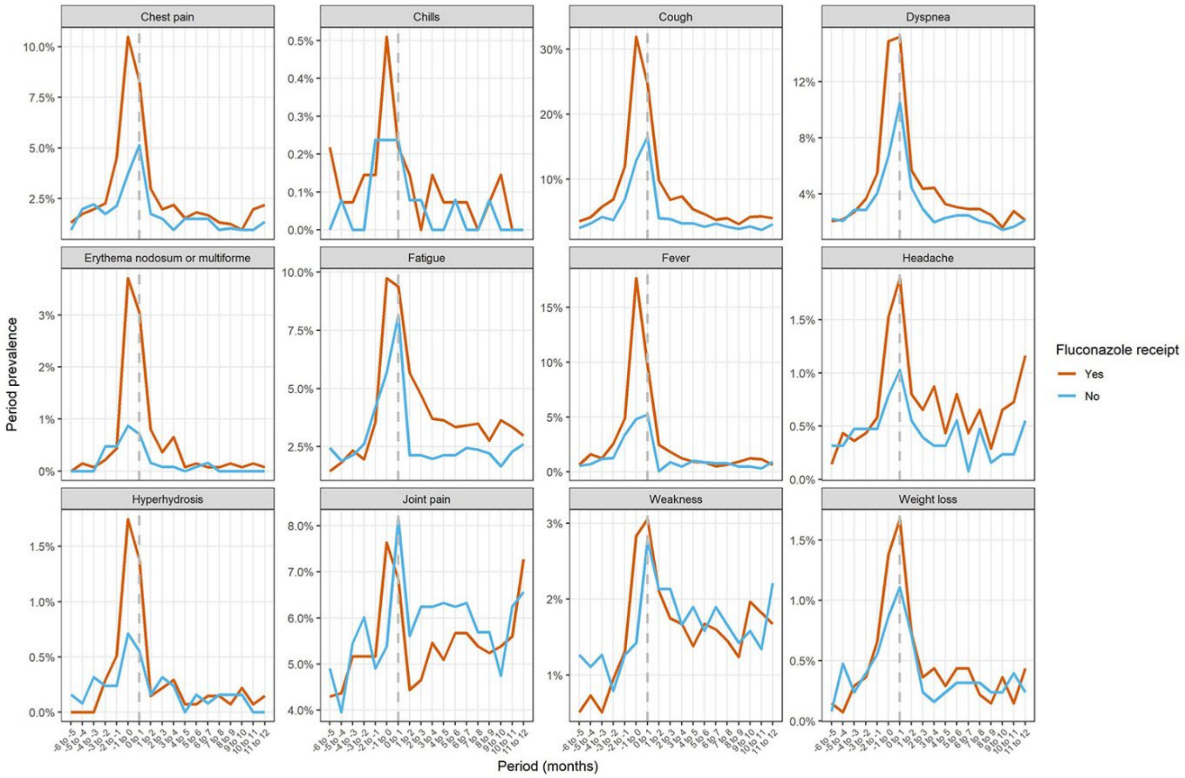

Appendix Figure 7. Period prevalence of selected coccidioidomycosis-associated symptoms among patients with coccidioidomycosis who received fluconazole versus those who did not — United States, July 2017–January 2023. Fluconazole receipt was defined as receipt of a ≥30-day supply. The index period (0–29 days after index date) is shown with a dotted line. Data for myalgia not shown due to prevalence <1%.

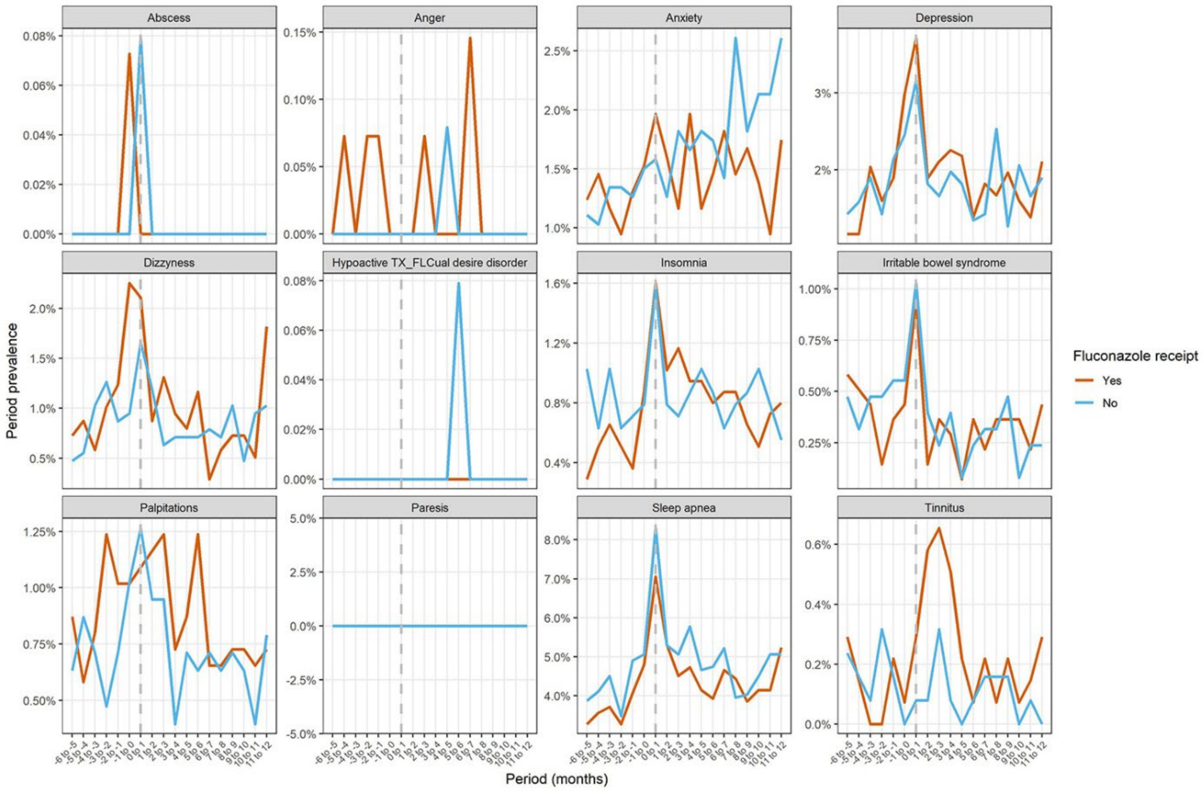

Appendix Figure 8. Period prevalence of selected IACCI-associated symptoms among patients with coccidioidomycosis for patients who received fluconazole versus those who did not — United States, July 2017–January 2023. Fluconazole receipt was defined as receipt of a ≥30-day supply. The index period (0–29 days after index date) is shown with a dotted line. Abbreviations: IACCI = infection-associated chronic conditions and illnesses. Anger, hypoactive sexual desire disorder, and paresis are not shown because prevalence was <0.1% in all periods.

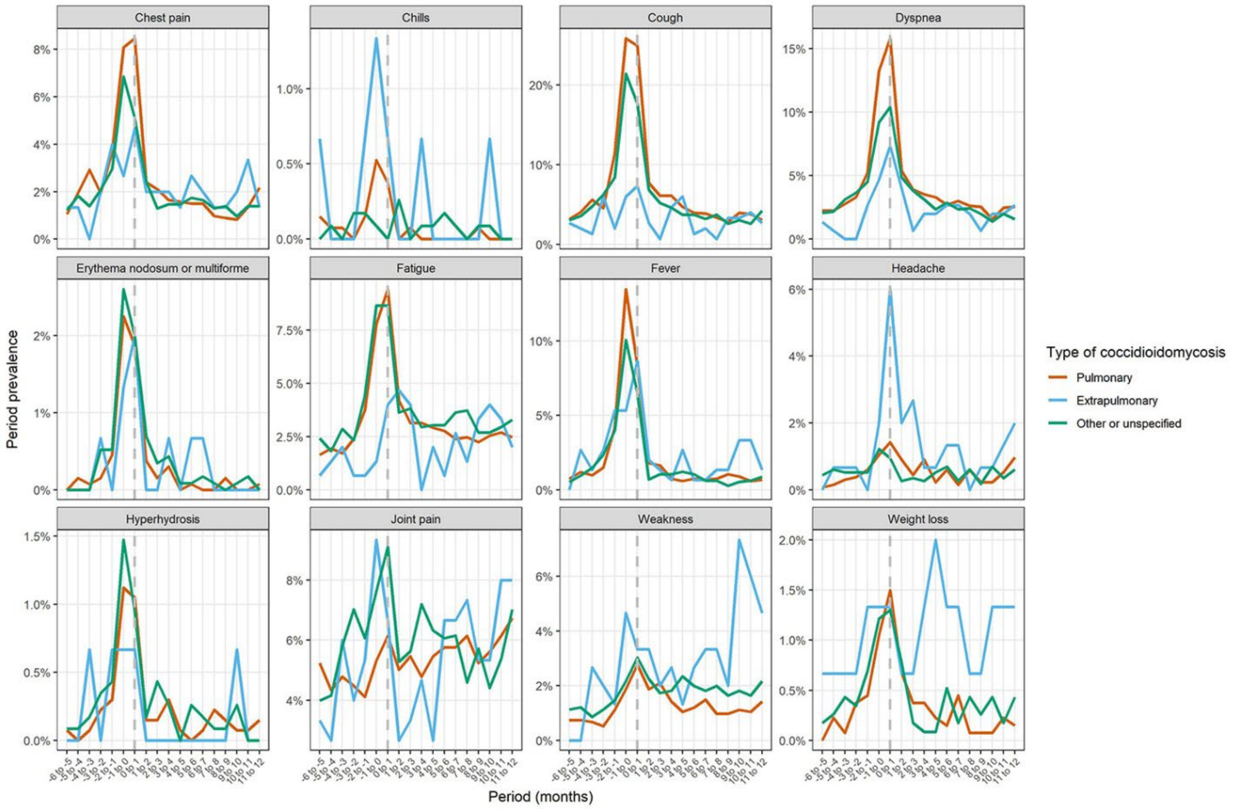

Appendix Figure 9. Period prevalence of selected coccidioidomycosis symptoms among patients with coccidioidomycosis by diagnosis type — United States, July 2017–January 2023. The index period (0–29 days after index date) is shown with a dotted line. Abbreviations: cocci = coccidioidomycosis. Data for myalgia not shown due to prevalence <1%.

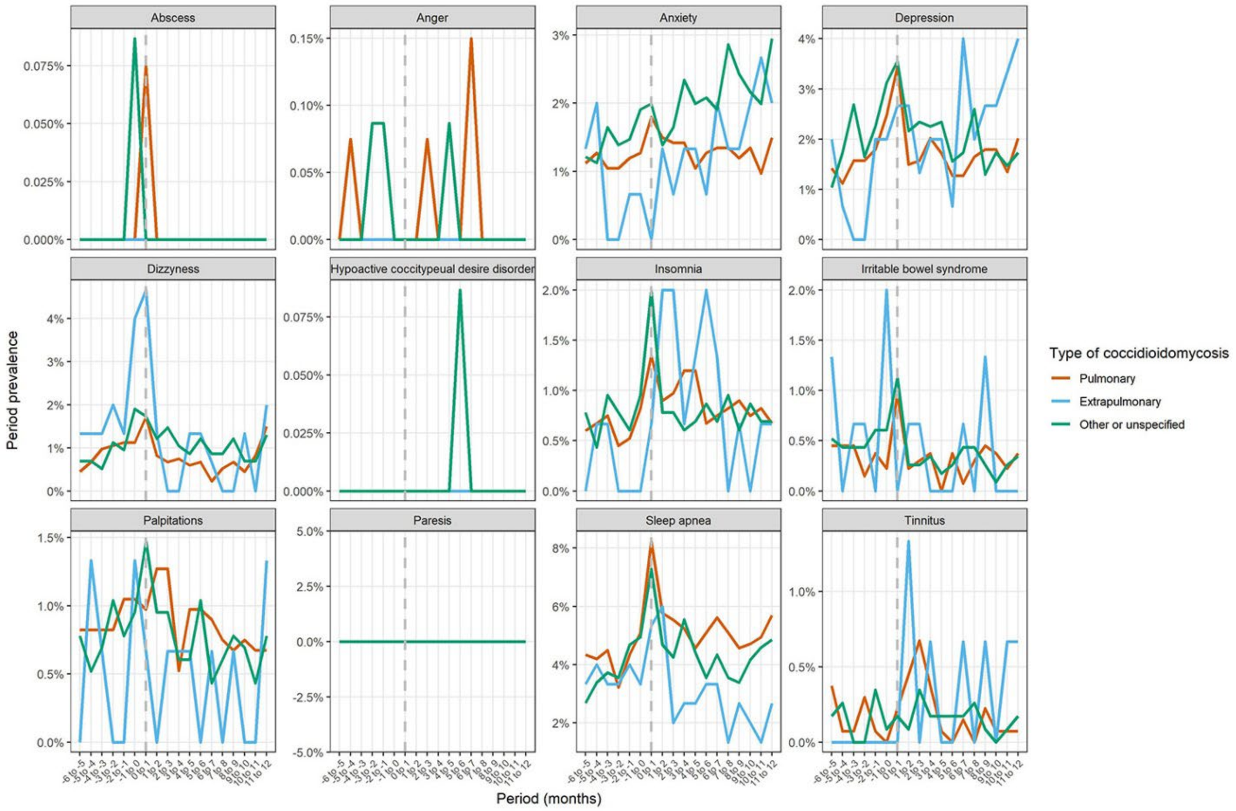

Appendix Figure 10. Period prevalence of selected IACCI-associated symptoms among patients with coccidioidomycosis by diagnosis type — United States, July 2017–January 2023. The index period (0–29 days after index date) is shown with a dotted line. Abbreviations: IACCI = infection-associated chronic conditions and illnesses. Anger, hypoactive sexual desire disorder, and paresis are not shown because prevalence was <0.1% in all periods.
